# Supplementary material for: Therapeutic potential of the secreted Kazal-type serine protease inhibitor SPINK4 in colitis
Source: Nat Commun. 2024 Jul 12;15:5874. doi: 10.1038/s41467-024-50048-y (PMC11245600; doi:10.1038/s41467-024-50048-y)
Supplement: Supplementary file 1 — Supplementary Information [file 41467_2024_50048_MOESM1_ESM.pdf]

Supplementary Information:

Therapeutic potential of the secreted Kazal-type serine protease inhibitor SPINK4 in colitis

Ying Wang, Jing Han, Guang Yang, Shuhui Zheng, Gaoshi Zhou, Xinjuan Liu, Xiaocang Cao, Guang Li, Bowen Zhang, Zhuo Xie, Li Li, Mudan Zhang, Xiaoling Li, Minhu Chen, Shenghong Zhang

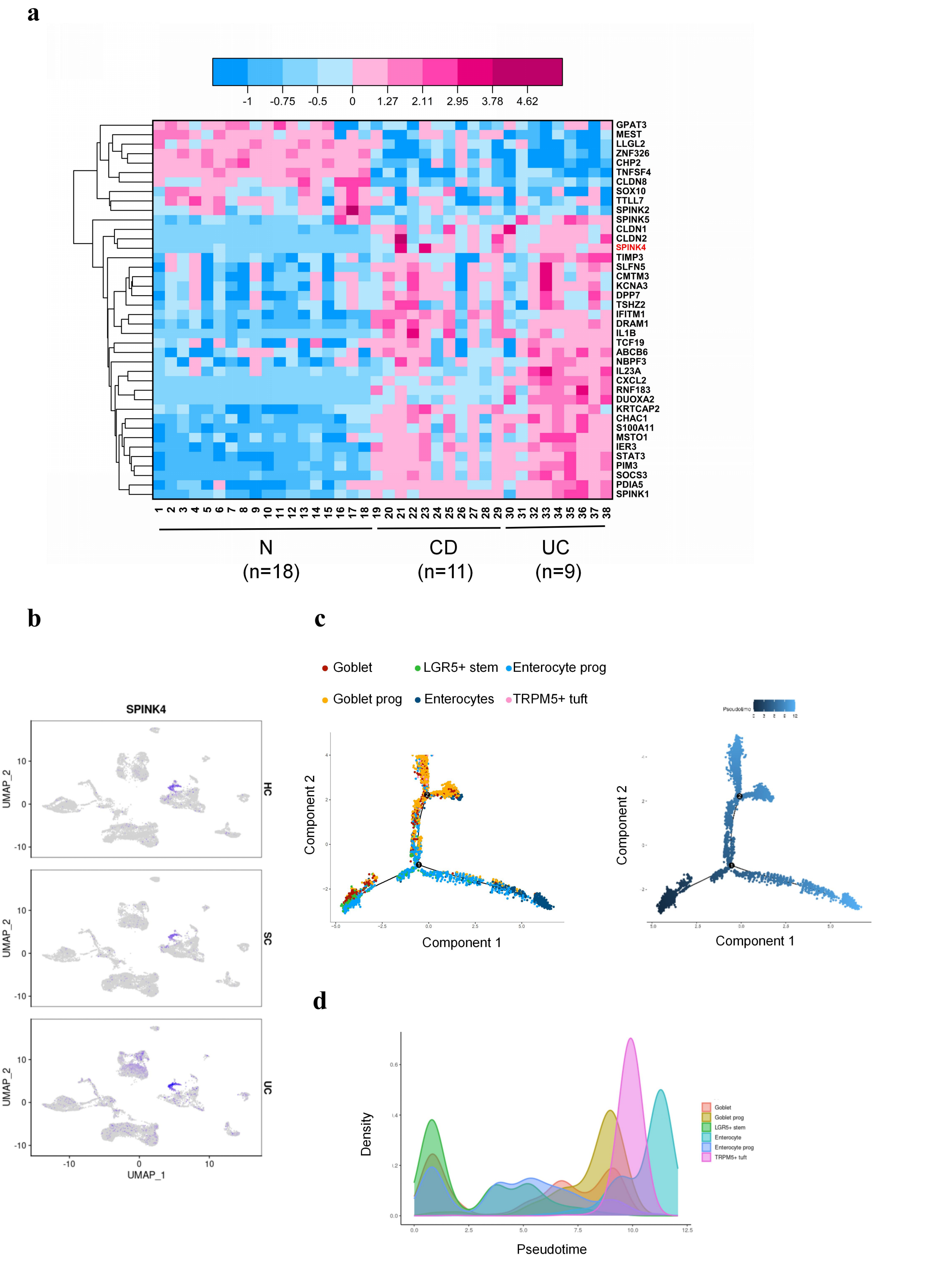

Supplementary Fig. 1: Transcriptional landscapes in IBD patients.

a Heatmap profile of RNA sequencing (RNA-seq) results from healthy controls (n = 18), CD

patients (n = 11), and UC patients (n = 9). Each row represents a specific RNA. Samples located at each column are presented with partial RNA expression after normalization. The color scale indicates different levels of RNA. Pink indicates high expression, while blue indicates low expression. *SPINK4* is indicated in red. **b** UMAP plot showing *SPINK4* located at GCs in healthy controls (HC), uninflamed regions from UC patients (SC), and inflamed regions from UC patients (UC) groups. **c** Left panel: differentiation trajectories in epithelial cells revealed using scRNA-seq in pseudotime order. Right panel: color scale ranges from black to blue, based on pseudotime analysis. **d** The dynamic epithelial generation with distinct cell types shown in a density plot.

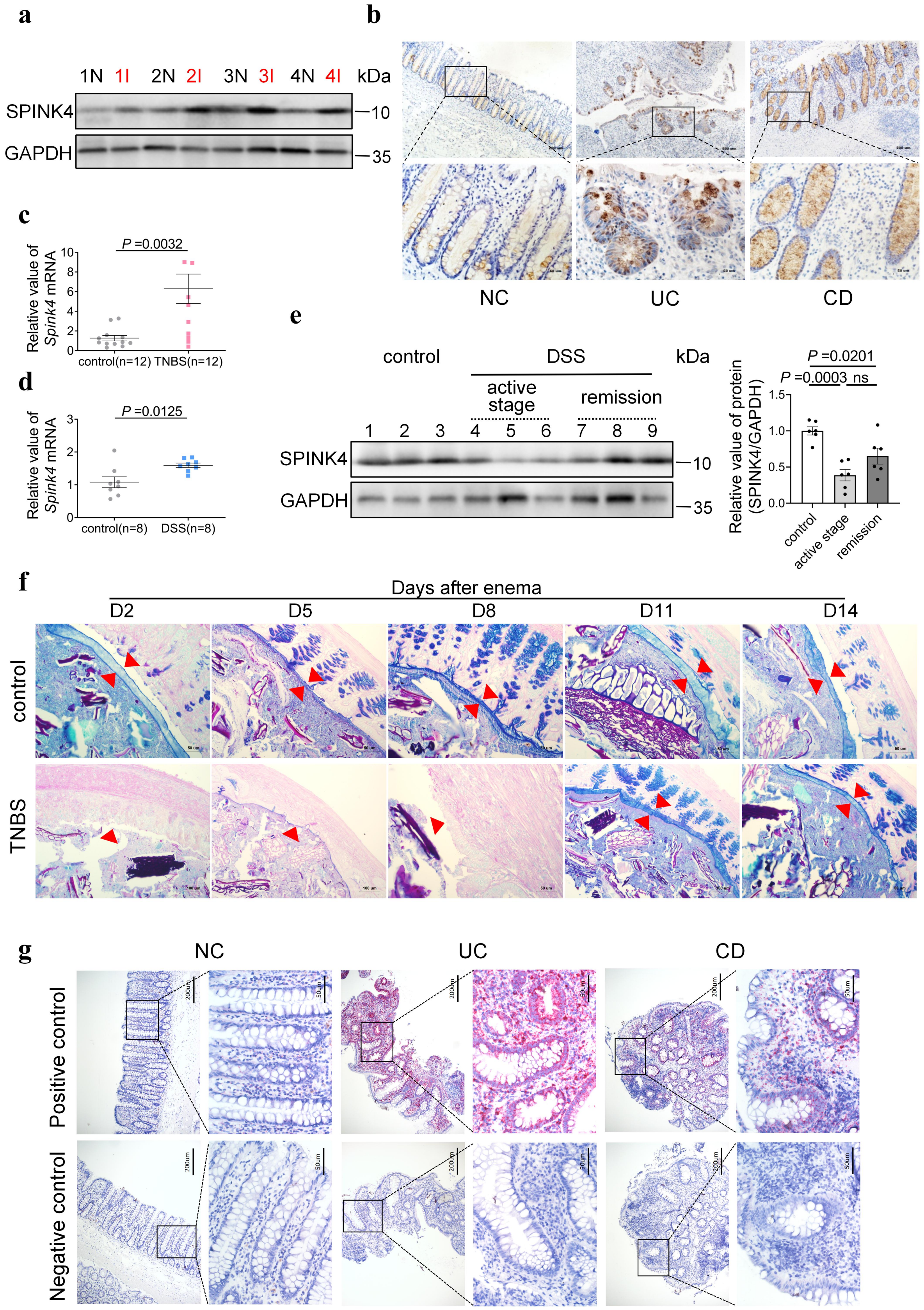

Supplementary Fig. 2: SPINK4 level is positively changed in colitis models along with

the mucus variation.

a Immunoblotting of surgical specimens from IBD patients (4 pairs of cases), including inflammatory lesion (red I) and relative normal tissue (black N). **b** Representative immunostaining targeting SPINK4 in tissues from surgical controls (NC) and IBD patients, including UC and CD. **c, d** Relative level of *Spink4* in TNBS (**c**) and DSS colitis models (**d**) at the chronic stage. **e** Differential expression of SPINK4 in DSS-induced colitis models at the active and remission stages via immunoblotting. Control (lines 1–3), DSS administration at the active stage (lines 4–6), and DSS administration at the remission stage (lines 7–9). **f** Typical performance of the inner mucus layer (red triangles) in the intestine of TNBS-induced colitis models or ethanol controls. **g** Compared with *SPINK4* localization, the negative and positive controls at the same view of the section presented using the Base Scope assay. Data are presented as the mean ± SEM. All tests were two-sided. Statistical significance was calculated using unpaired Student's *t*-test (**c**, **d**) and one-way analysis of variance (ANOVA) (**e**); n = 3, 4 biologically independent experiments (**a**, **e**). Source data are provided as a Source Data file.

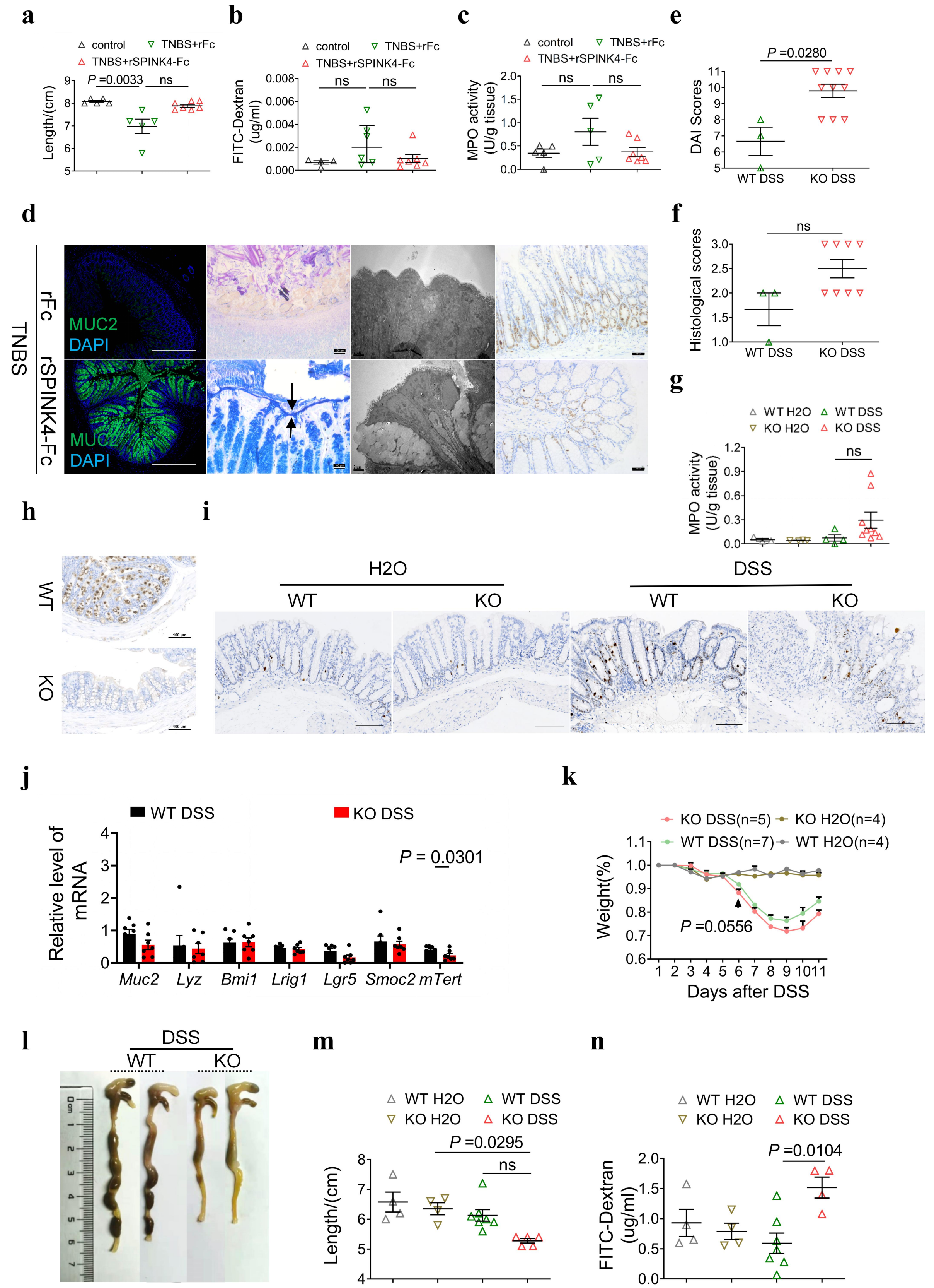

Supplementary Fig. 3: SPINK4 promotes GCs differentiation and influences epithelial

proliferation *in vivo*.

a–**c** Quantified analysis of the colonic length (**a**) and permeabilities assessed via FD4 (**b**) and tissue inflammation evaluated via MPO activity (**c**) in the following groups: ethanol control group (control), TNBS administration with rFc group (TNBS + rFc), and TNBS administration with rSPINK4-Fc group (TNBS + rSPINK4-Fc). **d** Intestinal images from rSPINK4-Fc or rFc treatment groups with TNBS administration obtained via immunofluorescence targeting MUC2 (green) with DAPI (blue) counterstaining, AB/PAS staining (arrow points to the mucus layer), TEM test for secretory vesicles from GCs, and immunohistochemistry test for detecting Ki67 levels. Unlabeled scale bar: 100 μm. **e**–**g** Inflammatory status is assessed using the DAI scores (**e**), histological scores (**f**), and MPO activity (**g**). **h** The representative images from WT and cKO mice targeting SPINK4. Scale bar: 100 μm. **i** Diminished proliferation measured by the Ki67-positive cell number in the cKO mice compared with WT mice. Scale bar: 100 μm. **j** Relative levels of biomarkers for GCs (*Muc2*), Paneth cells (*Lyz*), and stem cells (*Bmi1*, *Lrig1*, *Lgr5*, *Smoc2*, *mTert*) in WT and cKO mice. **k**–**n** Results from female mice, including weight loss (**k**), macroscopic morphology (**l**), length of colon (**m**), and intestinal permeabilities (**n**), obtained similarly in the co-housed control mice administrated H_2O (WT H2O, n = 4), cKO mice administrated H_2O (KO H2O, n = 4), co-housed control mice administrated DSS solution (WT DSS, n = 7), and cKO mice administrated DSS solution (KO DSS, n = 5). Data are presented as the mean ± SEM. All tests were two-sided. Statistical significance was calculated using unpaired Student's *t*-test (**j**) and one-way ANOVA (**d**, **k**, **n**). Kruskal-Wallis test (**a**, **b**, **g**, **m**) and Mann-Whitney U test (**e**, **f**) were performed on non-normal data; n =3–7 biologically

independent experiments (**b**, **c**, **g**). Source data are provided as a Source Data file.

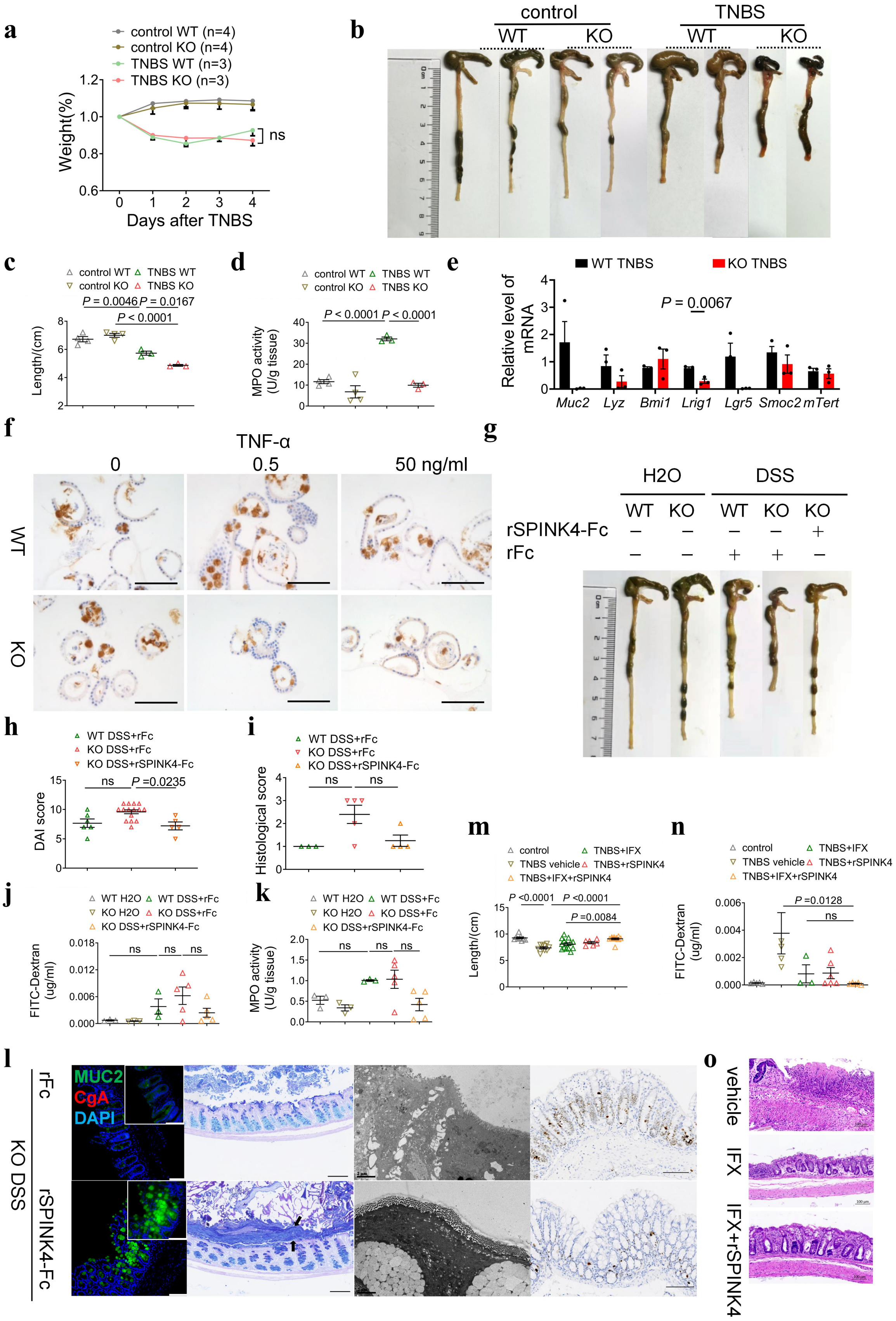

Supplementary Fig. 4: rSPINK4 reverses the exacerbation of colitis and dysfunction of

GCs induced by *Spink4*-knockout in vivo and in vitro.

a–**d** The assessment of disease activity by weight loss (**a**), appearance and length of the large intestine (**b, c**), and MPO activity (**d**) is demonstrated in the TNBS-induced colitis models with SPINK4 depletion. Mice were divided into 4 groups namely co-housed control mice administrated ethanol solution by enema (control WT, n = 4), cKO mice administrated ethanol solution by enema (control KO, n = 4), co-housed control mice administrated TNBS solution by enema (TNBS WT, n = 3), and cKO mice administrated TNBS solution by enema (TNBS KO, n =3). **e** In accordance with mice given DSS administration, the relative value of biomarkers in GCs, Paneth cells, and ISCs is shown in TNBS-induced colitis models. **f** The section of organoid derived from WT and cKO mice were stained with MUC2 antibody under inflammatory conditions. Scale bar: 100 μm. **g** Typical graphic view of the colon in five groups. **h**–**k** Assessment of disease activity by DAI scores (**h**), histological scores (**i**), permeabilities (**j**) and MPO activity (**k**) was conducted following rSPINK4 treatment in the cKO colitis mice. **l** Images from cKO mice treated with rSPINK4-Fc or rFc under colitis conditions performed via double immunofluorescence for MUC2 (green) and CgA(red), AB/PAS staining (arrow points to mucus layer), TEM test for GCs, and immunohistochemistry for Ki67. Unlabeled scale bar: 100 μm. **m-o** Length (**m**), permeability (**n**) of the colon and histological assessment with higher magnification (**o**) after IFX or rSPINK4 treatment under colitic conditions. Data are presented as the mean ± SEM. All tests were two-sided. Statistical significance was calculated using unpaired Student's *t*-test (**e**) and one-way ANOVA (**a**, **c**, **d**, **h**, **j**, **k**, **m**). Kruskal-Wallis test was performed in non-normal distribution data (**i**, **n**); n =3–15 biologically independent experiments (**d**, **e**, **h**–**k**, **n**). Source

data are provided as a Source Data file.

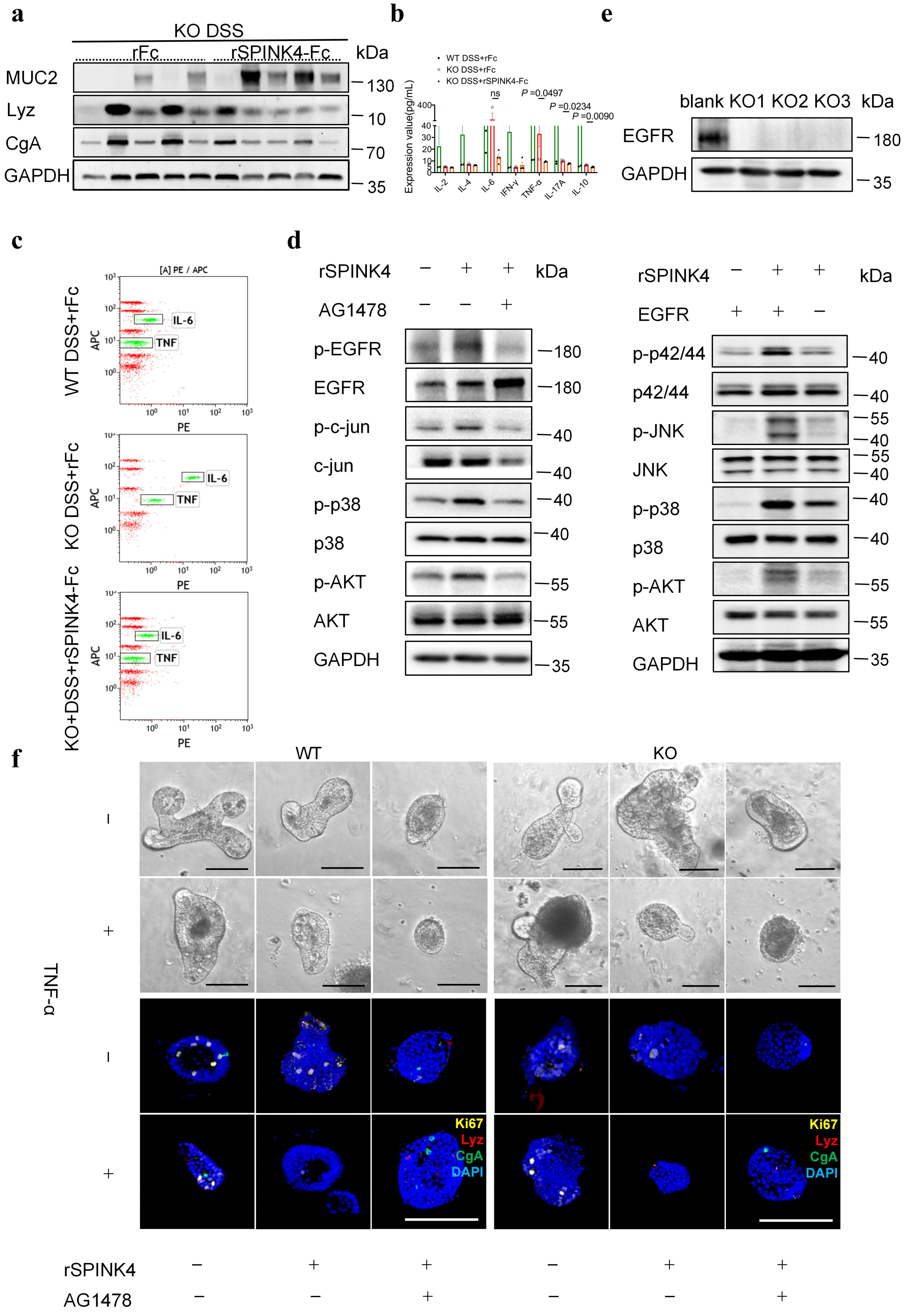

Supplementary Fig. 5: Mucus-inducing and inflammation-inhibiting functions of

rSPINK4 with the ability to influence proliferation in vitro.

a Quantification of GCs (MUC2), Paneth cells (Lyz), and enteroendocrine cells (CgA) under rSPINK4 treatment in cKO colitis mice. GAPDH was used as the loading control. **b, c** Expression (**b**) and flow cytometry pattern (**c**) of inflammatory factors including IL-2, 4, 6, 10, 17A; TNF-α; and IFN-γ. **d, e** The direct downstream target of the EGFR pathway was influenced with AG1478 (10 μM) in NCM460 cells (**d**) or EGFR deletion in LS174T cells (**e**). **f** Morphological image (upper panel) and expression (bottom panel) of proliferative cells (yellow: Ki67), Paneth cells (red: Lyz), and enteroendocrine cells (green: CgA) in the organoids derived from WT and cKO mice with or without the stimulation of TNF-α (50 ng/mL), rSPINK4 (100 ng/mL), and AG1478 (10 μM). Scale bar: 100 μm. Data are presented as the mean ± SEM. All tests were two-sided. Statistical significance was calculated using unpaired Student's *t*-test (**b**); n = 3 biologically independent experiments (**b**). Source data are provided as a Source Data file.

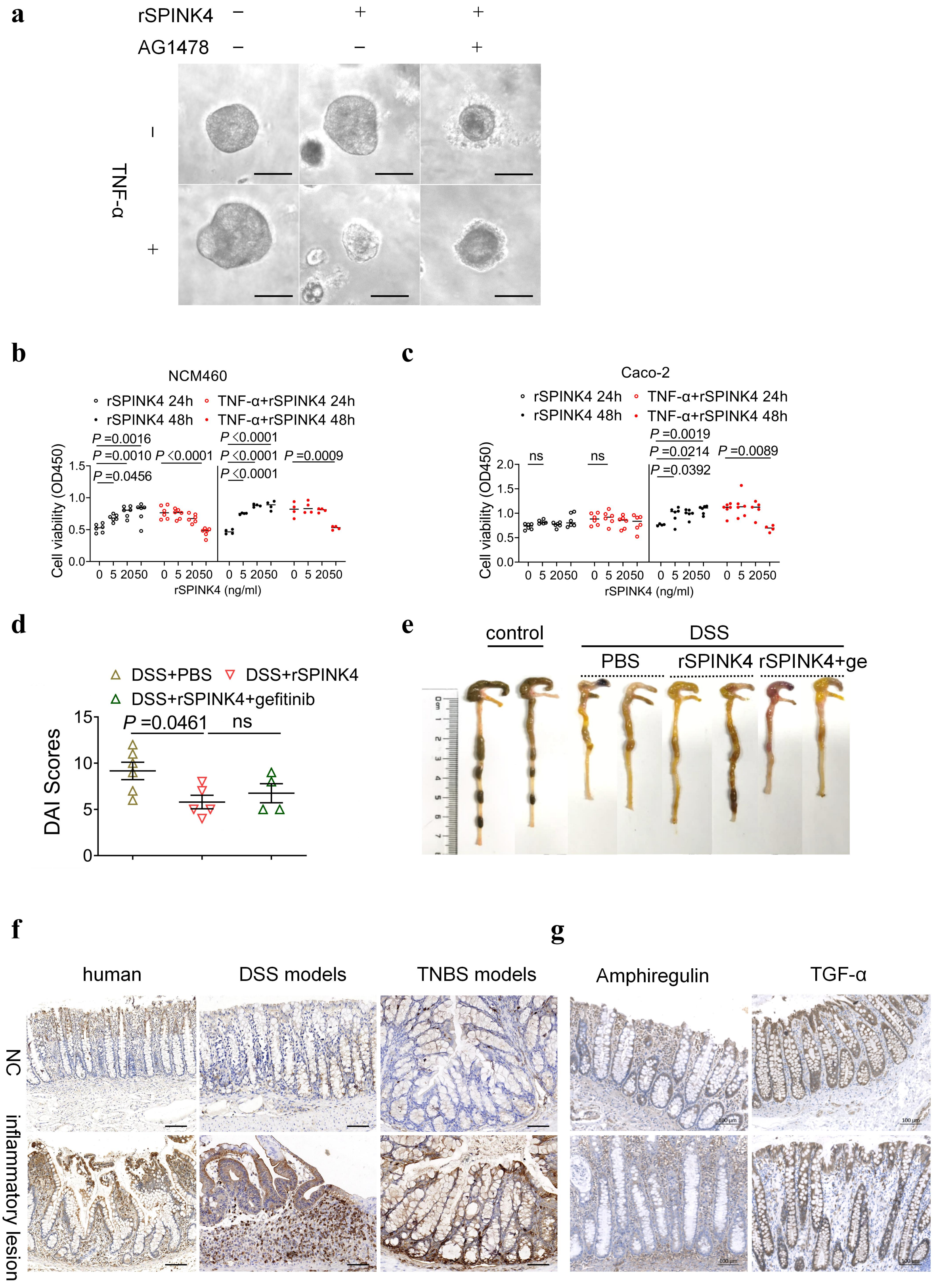

Supplementary Fig. 6: SPINK4 attributes to the colitis recovery without epithelium

proliferation in vitro.

a Organoid derived from CD patient shown via a morphological image under TNF-α (50 ng/mL), rSPINK4 (100 ng/mL), and AG1478 (10 μM) stimulation. Scale bar: 100 μm. **b, c** Cell viability measured using the CCK-8 test under normal or inflammatory conditions (50 ng/mL TNF-α) in NCM460 (**b**) and Caco-2 (**c**) cells. **d, e** Evaluation of disease activity was performed using the DAI scores (**d**) and appearance of the colon (**e**) in DSS-induced colitis mice following administration of rSPINK4 and gefitinib. **f, g** Immunohistochemical staining of phosphorylated EGFR(**f**) and classical ligands of EGFR(**g**) in colitis. Scale bar: 100 μm. Data are presented as the mean ± SEM. The Statistical tests were two-sided using one-way ANOVA (**b**–**d**); n =4–6 independent experiments (**b**–**d**). Source data are provided as a Source Data file.

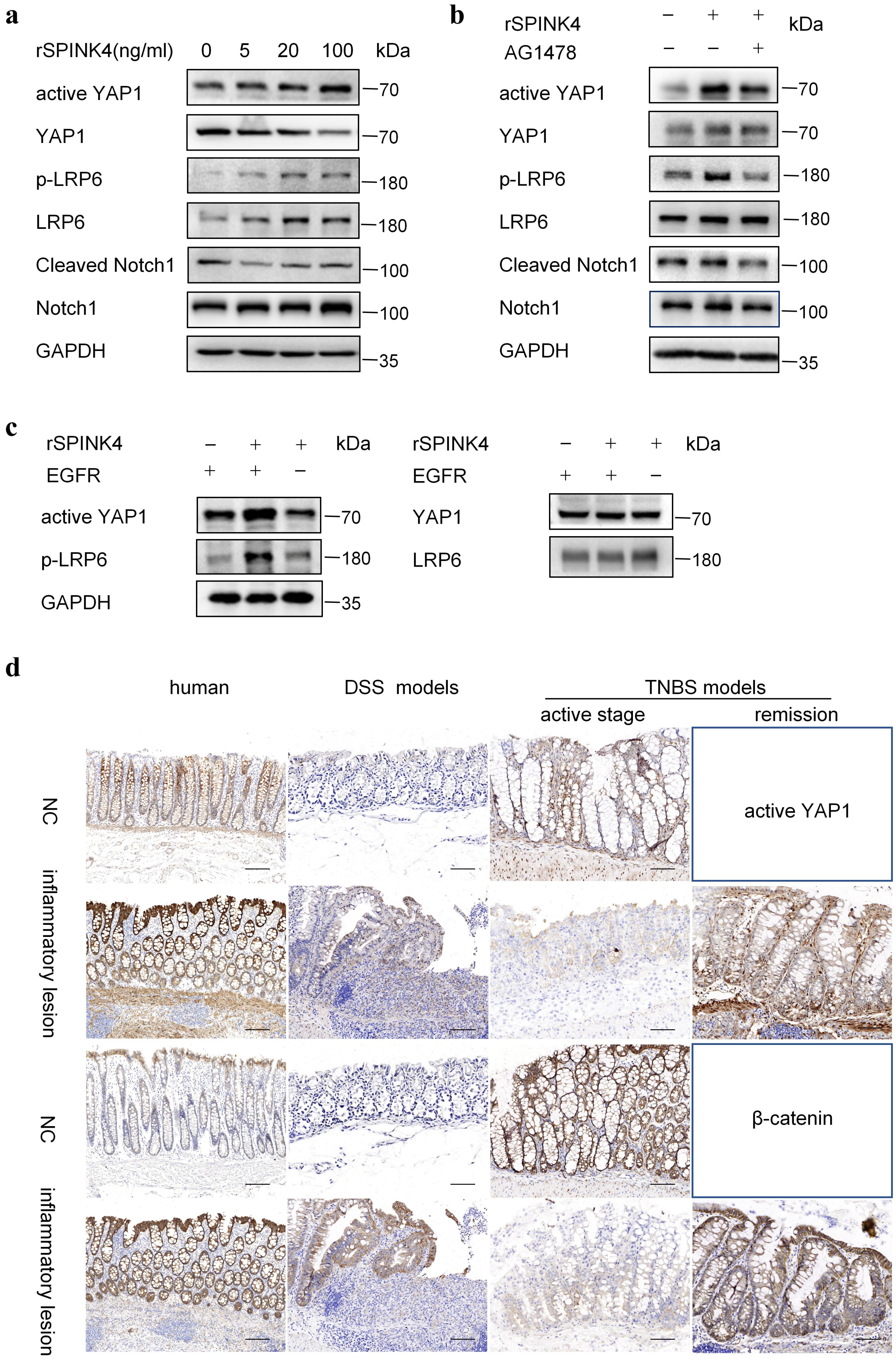

Supplementary Fig. 7: Hippo and Wnt/β-catenin pathways play an important role

downstream to EGFR.

a Hippo and Wnt pathways were influenced by rSPINK4 concentration in NCM460 cells. GAPDH was used as the loading control. **b, c** AG1478 (10 μM), an inhibitor of tyrosine kinase (**b**) and EGFR deficiency (**c**) reversed the influence exerted by 20 ng/mL rSPINK4. **d** Expression of active YAP1 and β-catenin (the key factor participating in the Wnt pathway) in IBD and colitis including different stages. Scale bar: 100 μm. Source data are provided as a Source Data file.

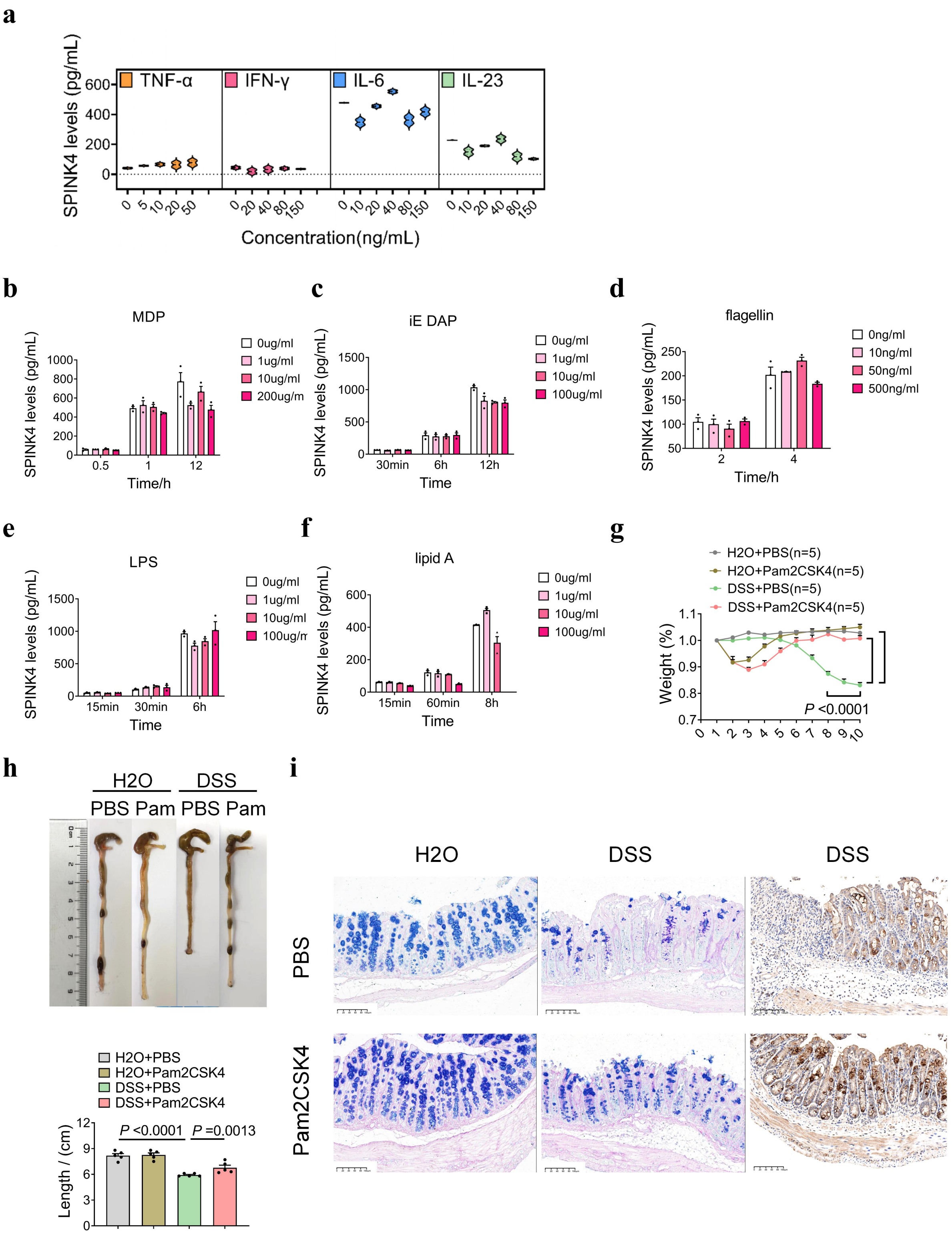

Supplementary Fig. 8: Irrelevant origination of SPINK4 in GCs.

a Relative level of *SPINK4* under the stimulation of TNF-α, IFN-γ, IL-6, and IL-23 for 24 h using ELISA test. **b**–**f** The other elements (**b**: MDP, **c**: iE DAP, **d**: flagellin, **e**: LPS, **f**: Lipid A) from the microbiota are irrelevant to *SPINK4* expression. **g, h** The body weight (**g**) and colonic length (**h**) were evaluated in the diverse groups namely control group (H2O + PBS, n=5), control with Pam2CSK4 group (H2O+ Pam2CSK4, n=5), DSS administration with PBS group (DSS + PBS, n=5), DSS administration with Pam2CSK4 group (DSS + Pam2CSK4, n=5). **i** Representative images of the colon from these groups presented with AB-PAS staining (left) and SPINK4 immunostaining (right). Data are presented as the mean ± SEM. Statistical tests were two-sided using one-way ANOVA (**a**–**f**, **g**) and Kruskal-Wallis test (**h**); n =3–5 independent experiments (**a**–**f**, **g**, **h**). Source data are provided as a Source Data file.

Supplementary Table 1. Sensitivity (Se) and specificity (Spe) of SPINK4 and other indices for clinical or endoscopic evaluation.

		Cut-off	AUROC	Se	Spe	*p* value
Clinical assessment	SPINK4	1687.94	0.651	61	66	0.01
	CRP	3.83	0.741	78	64	<0.001
	ESR	25.5	0.575	50	66	0.199
	PLT	317	0.64	53	77	0.016
Endoscopic evaluation	SPINK4	1894.09	0.729	49	93	0.001
	CRP	4.69	0.659	62	72	0.017
	ESR	15.5	0.581	62	65	0.225
	PLT	222.5	0.635	86	38	0.042

The tests were two-sided.

Supplementary Table 2. Antibodies used for western blotting in this study.

Antibodies	Concentration	Corporation	Product code	Reactive species
SPINK4	0.736111111	genetex	custom services	mouse
SPINK4	0.736111111	abcam	ab175929	human
GAPDH	3.513888889	proteintech	10494-1-AP	human, mouse
β-actin	3.513888889	proteintech	66009-1-Ig	human, mouse
MUC2	0.736111111	abcam	ab272692	human, mouse
Lyz	1: 10000	abcam	ab108508	human, mouse
CgA	0.388888889	Santa cruz	sc-393941	human, mouse
p-EGFR (phospho-EGFR Y1068)	0.736111111	abcam	ab40815	human, mouse
EGFR	1.430555556	proteintech	66455-1-Ig	human, mouse
p-AKT (phospho-AKTSer473)	1.430555556	CST	#4060	human, mouse
AKT	0.736111111	CST	#9272	human, mouse
p-p38 (phospho-p38 MAPK Thr180/Tyr182)	0.736111111	CST	#4511	human, mouse
p38	0.736111111	CST	#8690	human, mouse
p-p42/44 (phospho-p44/42 MAPK (Erk1/2) Thr202/Tyr204)	1.430555556	CST	#4370	human, mouse
p42/44	1.430555556	CST	#4696	human, mouse
p-c-jun (phospho-c-Jun Ser73)	0.736111111	CST	#3270	human, mouse
c-jun	0.736111111	CST	#9165	human, mouse
p-JNK (phospho-SAPK/JNK Thr183/Tyr185)	0.736111111	CST	#4668	human, mouse
JNK	0.736111111	CST	#9252	human, mouse
His-tagged	3.513888889	proteintech	66005-1-Ig	human, mouse
FLAG-tagged (Anti-DDDDK tag)	1.430555556	proteintech	20543-1-AP	human, mouse
active YAP1	0.736111111	abcam	ab205270	human, mouse
YAP1	0.736111111	CST	#14074	human, mouse
p-LRP6 (phospho-LRP6 Ser1490)	0.736111111	CST	#2568	human, mouse
LRP6	0.736111111	CST	#3395	human, mouse
Cleaved Notch1	0.736111111	CST	#4147	human, mouse
Notch1	0.736111111	CST	#3608	human, mouse
